# Podocyte and endothelial injury in focal segmental glomerulosclerosis: an ultrastructural analysis

**DOI:** 10.1007/s00428-015-1821-9

**Published:** 2015-08-13

**Authors:** Sekiko Taneda, Kazuho Honda, Mayuko Ohno, Keiko Uchida, Kosaku Nitta, Hideaki Oda

**Affiliations:** Department of Pathology, Tokyo Women’s Medical University, Tokyo, Japan; Department of Internal Medicine, Kidney Center, Tokyo Women’s Medical University, Tokyo, Japan; Department of Anatomy, School of Medicine, Showa University, Tokyo, Japan

**Keywords:** Electron microscopy, Focal segmental glomerulosclerosis, Foot process effacement, Podocyte detachment, Subendothelial widening

## Abstract

Podocyte injury contributes to the development of focal segmental glomerulosclerosis (FSGS). Endocapillary hypercellularity, which is one of the pathological characteristics of FSGS, suggests that glomerular endothelial injury may also be involved in the pathogenesis of FSGS. In electron micrographs of patients with FSGS (*n* = 43), we conducted morphometric measurements of foot process width (FPW) and podocyte detachment (PD) as markers of podocyte injury and subendothelial widening (SW) of the glomerular basement membrane as a marker of endothelial injury and compared them to those in patients with minimal change nephrotic syndrome (MCNS; *n* = 11) and control kidney donors (*n* = 5). Associations between ultrastructural and clinical parameters were analyzed according to the FSGS variants defined by the Columbia classification. FPW was significantly higher in the FSGS group than that in the MCNS and control groups, particularly in the collapsing, tip, and cellular variants of FSGS. Percentage of glomerular basement membrane (GBM) length showing PD and SW was significantly increased in the FSGS group, especially in the collapsing, cellular, and not otherwise specified variants. In FSGS, FPW was inversely correlated with disease duration, but not with proteinuria. Finally, the percentage of GBM length with SW significantly correlated with clinical parameters indicative of poor prognosis, such as lower remission rate and lower estimated glomerular filtration rate at the final observation. Quantitative measurement of podocyte and endothelial injury by electron microscopy might be useful for evaluating histological activity and predicting prognosis in FSGS.

**Electronic supplementary material:**

The online version of this article (doi:10.1007/s00428-015-1821-9) contains supplementary material, which is available to authorized users.

## Introduction

Focal segmental glomerulosclerosis (FSGS) is morphologically defined by various histologic patterns of segmental glomerular lesions involving tuft capillary collapse, podocyte hyperplasia and hypertrophy, endocapillary hypercellularity, sclerosis, and hyaline deposits. Podocytes are a major contributor in the pathogenesis of FSGS [9, 25]. Ultrastructural abnormalities of podocytes, such as foot process effacement [9, 25], vacuolization [32], cytoplasmic droplets [25], microvillous transformation [21], and podocyte detachment (PD) from the glomerular basement membrane (GBM) [9, 25], have been reported in FSGS. Although these alterations are not specific for FSGS, various morphological alterations of podocytes suggest their importance in the pathogenesis of FSGS.

In 2004, D’Agati et al. proposed the Columbia classification that identifies the following five histologic variants of FSGS: collapsing (COL), tip (TIP), cellular (CEL), perihilar (PH), and not otherwise specified (NOS) [3]. Among them, the CEL variant is characterized by the presence of endocapillary hypercellularity, intracapillary aggregates of endothelial cells, foam cells, and infiltrating leukocytes, which results in capillary occlusion [3]. The significance of this lesion is unknown, but an immunohistochemical study has demonstrated that the glomerular capillaries of the segmental lesion are negative for the endothelial marker CD31 [25], which suggests that segmental lesion with endocapillary hypercellularity may be a sign of endothelial damage and endothelial injury may be involved in the development of FSGS. Ultrastructural alterations representing endothelial damage include endothelial swelling [7], decrease of the endothelium or of its fenestrations [30], and subendothelial widening (SW) of GBM [20]. SW is characterized by the presence of a lucent zone in the lamina rara interna and it is the most frequent glomerular lesion attributed to endothelial damage [17]. SW has been reported in human cases of endothelial damage such as transplant glomerulopathy, thrombotic microangiopathy, or hemolytic uremic syndrome, in which evolving chronicity is occasionally accompanied by segmental glomerular sclerosis [11, 16, 19, 26]. Therefore, it is quite conceivable that podocyte and glomerular endothelial injury are involved in the development of FSGS. A few morphometric studies have evaluated podocyte abnormalities in FSGS [5, 31]. They focused on foot process effacement by quantitating the foot process width (FPW), but other morphological alterations of podocyte or endothelial damage have rarely been examined. In this study, we morphometrically examined the three parameters of FPW, PD, and SW ultrastructurally, because a quantitative approach was feasible in the electron micrographs obtained from patients with FSGS. We compared them with those in patients with minimal change nephrotic syndrome (MCNS), which is widely accepted as podocytopathy. The associations between ultrastructural and clinical parameters were evaluated among FSGS variants defined by the Columbia classification. Kidney transplant donors served as controls.

## Subjects and methods

### Patients and controls

Forty-three patients with biopsy-proven FSGS cases were enrolled in this study. We excluded cases who were diagnosed with other primary glomerular diseases, collagen diseases, or diabetes, as well as cases associated with secondary FSGS from various causes, such as obesity (body mass index >28 kg/m^2^), renal atrophy, unilateral renal agenesis, reflux nephropathy, family histories of renal diseases, or malignancies [3]. Therefore, all cases were principally considered to have idiopathic FSGS. The following data were collected from the patients’ medical records: age, sex, disease duration, proteinuria (g/day), and estimated glomerular filtration rate (eGFR; mL/min/1.73 m^2^) at the time of biopsy. Proteinuria and eGFR were also evaluated at the final observation. The change in eGFR from the time of biopsy to the final observation was calculated and initiation of hemodialysis (HD) was also reviewed. For comparison, we used renal biopsy specimens from MCNS patients (*n* = 11) and zero-hour biopsy specimens from control renal transplant donors (*n* = 5) who showed no histologic abnormalities by light microscopy. Some cases enrolled in this study were also subjects in our previous study [29]. The study protocol and informed consent procedure were approved by the ethics committee of the Tokyo Women’s Medical University Hospital.

### Light microscopy and immunofluorescence microscopy

The renal tissues were stained with hematoxylin and eosin, periodic acid–Schiff, Masson’s trichrome, and periodic acid methenamine silver. FSGS cases were classified into the following five variants according to the Columbia criteria [3]: COL, TIP, CEL, PH, and NOS. Positive staining for segmental IgM and C3 was confirmed in cases of FSGS, whereas in MCNS subjects or control donor, immunostaining was not considered.

### Electron microscopic examination

Specimens for electron microscopy were obtained at the time of biopsy. The authors took eight randomly unbiased electron microscopic photographs of glomeruli in each case, at magnifications of ×4000, ×4500, ×5000, and ×6000, with two photographs at each magnification. The following three parameters were then measured with the image analysis software, ImageJ (National Institute of Health, Rockville, MD, USA): FPW, length of PD, and SW. FPW was measured according to a previously reported method [5].

**Fig. 1:**
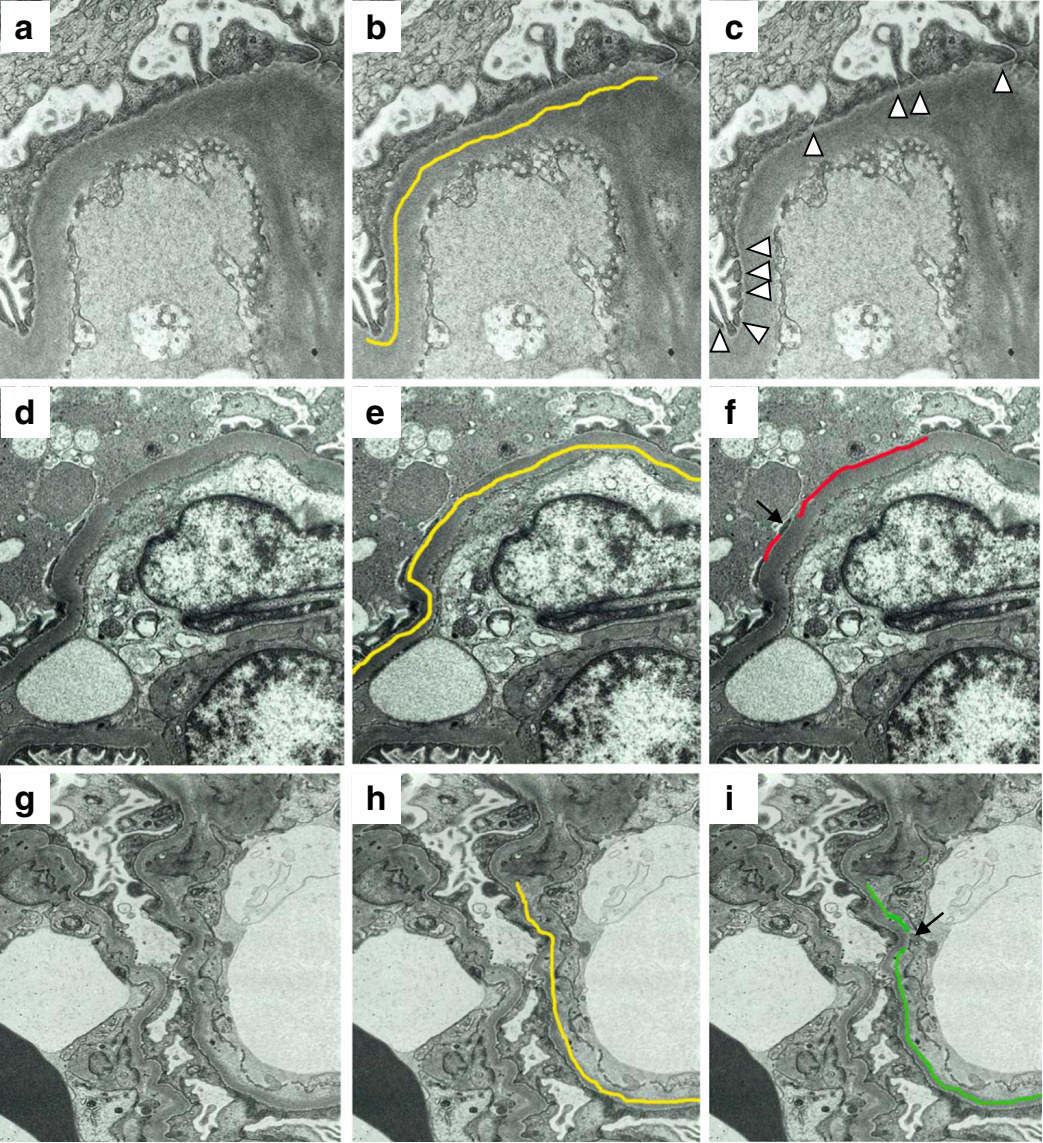
Electron micrographs (**a**–**i**), illustrating the measurement of the foot process width (FPW; **a**–**c**), the length of podocyte detachment (PD; **d**–**f**), and the subendothelial widening (SW; **g–i**) of the glomerular basement membrane in the kidneys that were obtained from patients with the tip (TIP; **a**–**c**), not otherwise specified (NOS; **d**–**f**) and cellular (CEL; **g**–**i**) variants of FSGS. **b**, **e**, **h** Length of the glomerular basement membrane (GBM) on electron microscopy determined by the morphometric analysis (*yellow line*). **c** An electron micrograph illustrating the separations of the foot processes (*arrowheads*). **f** An electron micrograph illustrating the measurement of the length of GBM with podocyte detachment by *two red lines*. A podocyte is intact in the area between the red lines (*arrow*). **i** An electron micrograph illustrating the measurement of the length of GBM with subendothelial widening by *two green lines*. GBM between the green lines is the area where subendothelial widening is not present (*arrow*)

In order to illustrate how the foot processes were counted and length of PD and SW was measured, electron microscopic images of TIP (for the FPW: Fig. 1a–c), NOS (for PD: Fig. 1d–f), and CEL variant (for SW: Fig. 1g–i) are shown in Fig. 1, because foot process effacement was found in all variants of FSGS including TIP variant, while PD and SW were observed in NOS and CEL variants more often than in other variants. Total length of GBM was measured (Fig. 1b), and the number of podocytic foot processes was counted in each GBM (Fig. 1c). A foot process was defined as cytoplasmic extensions, separated from adjacent foot processes by lateral membranes (Fig. 1c) [5]. Separations between foot processes were marked with arrowheads (Fig. 1c). For each patient, the mean FPW was calculated by dividing the total length of GBM by the total number of intervals between the foot processes.

In order to obtain GBM length showing PD or SW relative to the total analyzed length of GBM (% of GBM length with PD or SW, respectively), the total GBM length was measured (Fig. 1e, h), and the length of PD and SW was measured in all GBMs visualized on the electron micrographs (Fig. 1f, i). For each patient, an average percentage of GBM length with PD or SW was calculated by dividing the total length of PD or SW by the total length of GBM.

### Definitions

Complete remission (CR) was defined as proteinuria <0.3 g/24 h with a normal eGFR range (≥90 mL/min/1.73 m^2^) at the final observation and/or with an improved eGFR change from baseline (increase more than +10 mL/min/1.73 m^2^). Partial remission (PR) was defined as proteinuria between 0.3 and 2.0 g/24 h, with a stabilized eGFR change between −10 and +10 mL/min/1.73 m^2^. No response (NR) was defined as proteinuria ≥2.0 g/24 h with a progressive decrease in eGFR from baseline (below less than −10 mL/min/1.73 m^2^). Renal failure was defined as the clinical state requiring maintenance HD.

### Statistical analysis

The data are presented as the mean ± standard error of the mean, geometric means with 95 % confidence intervals, or mean as appropriate according to the data distribution. For statistical analyses, Student’s *t* tests, Wilcoxon’s summed rank tests or Kruskal–Wallis tests, Fisher’s exact tests, and multivariate linear regression analyses were used as appropriate. To elucidate associations among the clinicopathological parameters, Spearman correlation coefficients (*r*) were calculated. A multivariate analysis was performed in a forward stepwise fashion to determine the relationship between each of the ultrastructural parameters and clinical variables that were identified as being significant at *P* < 0.05 by univariate analysis. The significance level was set at a two-sided *P* value of <0.05. The analysis was performed with SPSS 14.0 for windows (SPSS Inc. Japan, Tokyo, Japan).

## Results

### Clinical characteristics of patients with FSGS and MCNS

Clinical features of the FSGS and MCNS groups are summarized in Table 1. Patients in the FSGS group demonstrated a significantly longer disease duration, particularly in NOS and PH variants, compared to those in the MCNS group (*P* < 0.01). At the time of biopsy, the eGFR in the FSGS group was significantly lower than that in the MCNS group (*P* < 0.01), and the degree of proteinuria was significantly lower in NOS and PH variants compared to other variants of FSGS or to the MCNS group (NOS or PH variant vs. MCNS group; *P* < 0.05). The controls had no proteinuria, with a mean eGFR of 91.2 ± 9.2 mL/min/1.73 m^2^.

**Table 1:** Clinical characteristics of patients with FSGS and MCNS

	FSGS						MCNS	*P* value^a^
	All	COL	TIP	CEL	PH	NOS		
Case number	43	5 (11.6 %)	14 (32.6 %)	8 (18.6 %)	4 (9.3 %)	12 (27.9 %)	11	
Age	38.8 ± 2.4	34.0 ± 3.2	41.2 ± 5.0	36.6 ± 2.7	34.8 ± 5.1	39.4 ± 4.8	34.6 ± 3.8	NS
Male/female	23/20	2/3	7/7	4/4	3/1	7/5	7/4	NS
Disease duration (months)	70.8 ± 14.8	12.5 ± 2.5	8.1 ± 3.5	20.6 ± 6.4	96.0 ± 44.3	102.1 ± 26.0	1.1 ± 0.1	<0.001
Initial urinary protein (g/day)	6.5 ± 1.0	6.7 ± 1.5	9.0 ± 2.1	10.3 ± 2.6	3.0 ± 1.4	2.0 ± 0.6	6.3 ± 0.8	NS
Initial eGFR (mL/min/1.73 m^2^)	53.4 ± 3.8	55.0 ± 16.1	52.6 ± 6.2	53.1 ± 22.8	54.3 ± 8.6	53.4 ± 8.5	82.5 ± 6.6	<0.01
Follow-up duration (years)	5.3 ± 0.4	4.0 ± 1.1	6.8 ± 0.7	4.4 ± 0.8	5.5 ± 1.0	4.5 ± 0.8	4.1 ± 0.7	NS
Final urinary protein (g/day)	1.24 ± 0.29	1.48 ± 0.63	0.97 ± 0.47	2.08 ± 1.04	0.62 ± 0.08	1.14 ± 0.49	0.05 ± 0.05	<0.001
Final eGFR (mL/min/1.73 m^2^)	43.1 ± 4.3	22.7 ± 11.8	50.0 ± 7.8	41.2 ± 28.0	57.3 ± 8.6	40.0 ± 7.9	84.2 ± 4.7	<0.001
△eGFR (mL/min/1.73 m^2^)	−11.3 ± 4.9	−32.2 ± 10.0	−2.8 ± 10.4	−11.9 ± 11.6	3.0 ± 4.1	−13.4 ± 5.9	1.7 ± 4.2	NS
Remission (% of NR)	35.7	100	21.4	57.1	0	41.7	0	<0.05
Hemodialysis (%)	9/42 (21.4)	3/4 (75)	2/14 (14.3)	2/8 (25)	0/4 (0)	2/12 (16.7)	0/11 (0)	NS

Quantitative variables are mean ± standard error

*COL* collapsing variant, *TIP* tip variant, *CEL* cellular variant, *PH* perihilar variant, *NOS* not otherwise specified variant, *MCNS* minimal change nephrotic syndrome, *eGFR* estimated glomerular filtration rate, △*eGFR* eGFR at the final observation − eGFR at the time of biopsy, *NR* no response, *NS* not significant

^a^Analysis of variance between all FSGS vs MCNS, Student *t* test, or Fisher exact test

At final observation, the degree of proteinuria was higher and eGFR lower in the FSGS group than in the MCNS group (*P* < 0.05, irrespective of the histological variant). No significant difference was detected in the change in eGFR between the two groups, although the reduction in eGFR in the COL and NOS variants was significantly greater than that in the MCNS group (*P* < 0.05, Supplemental data 1). The percentage of NR was significantly higher in the FSGS group, particularly in the COL, CEL, and NOS variants, than that in the MCNS group (*P* < 0.05). In contrast, 79 % of patients with the TIP variant showed CR or PR, and all patients with the PH variant showed PR (data not shown). No significant difference was detected in the overall percentage of patients needing HD between the FSGS and MCNS groups, although 75 % of patients with the COL variant required HD..

### Representative figures of the FSGS variants

**Fig. 2:**
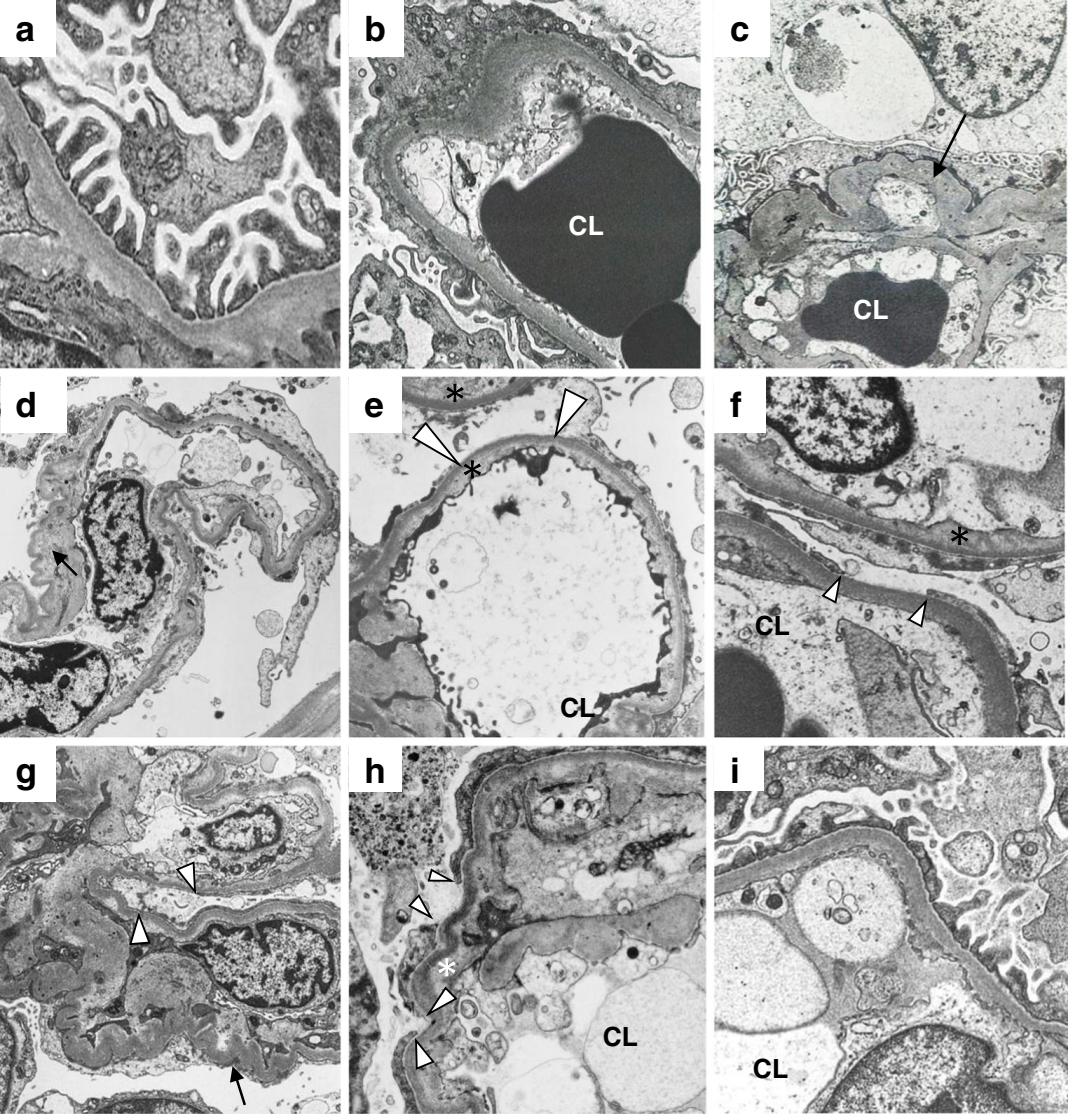
Representative electron micrographs of the kidneys from a control donor (**a**), a patient with minimal change nephrotic syndrome (**b**), and patients with each of the following variants of focal segmental glomerulosclerosis (FSGS): tip variant (**c**), collapsing variant (**d**, **e**), cellular variant (**f**), not otherwise specified variant (**g**, **h**), and perihilar variant (**i**). In the micrographs of control kidney, separations between the foot processes were located at regular intervals on the glomerular capillary (**a**). In the micrographs of the MCNS kidneys, effacement of the foot processes were marked (**b**). Foot process effacement was also marked in the micrographs from patients with the TIP (**c**) the COL (**d** and **e**), CEL (**f**), and NOS variants (**g** and **h**). Podocyte detachment and subendothelial widening were apparent in the micrographs from patients with COL, the CEL, and NOS variants (**d**, **e**, **f**, **g**, and **h**), but not in patients with PH variant (**i**) or in patients with MCNS (**b**). *CL*—capillary lumina, *arrowheads*—podocyte detachment, *asterisk*—subendothelial widening, *arrow*—collapse of glomerular capillary

Representative electron microscopic images of each variant of the FSGS, MCNS, and the control donor kidneys are shown in Fig. 2. In micrographs of the control kidney, separations between foot processes were located at regular intervals on the glomerular capillary (GC; Fig. 2a). In micrographs of MCNS kidneys, GCs showed marked effacement of foot processes (Fig. 2b). The GC showed marked effacement of foot processes in micrographs from patients with the TIP variant (Fig. 2c) as well as patients with the COL (Fig. 2d, e), CEL (Fig. 2f), and NOS variants (Fig. 2g, h). In micrographs from patients with the COL variant, parts of GCs were collapsed and showed PD and SW (Fig. 2d, e). Furthermore, partial PD and SW were apparent in patients with the CEL and NOS variants (Fig. 2g, h, and PD in patients with the NOS variant is also shown in Fig. 1d), but not in patients with other variants or with MCNS. Patients with the PH variant demonstrated preserved separations between foot processes, and slight effacement of foot processes was partially observed (Fig. 2i).

### Morphometric analysis of FPW and the percentage of PD and SW

**Fig. 3:**
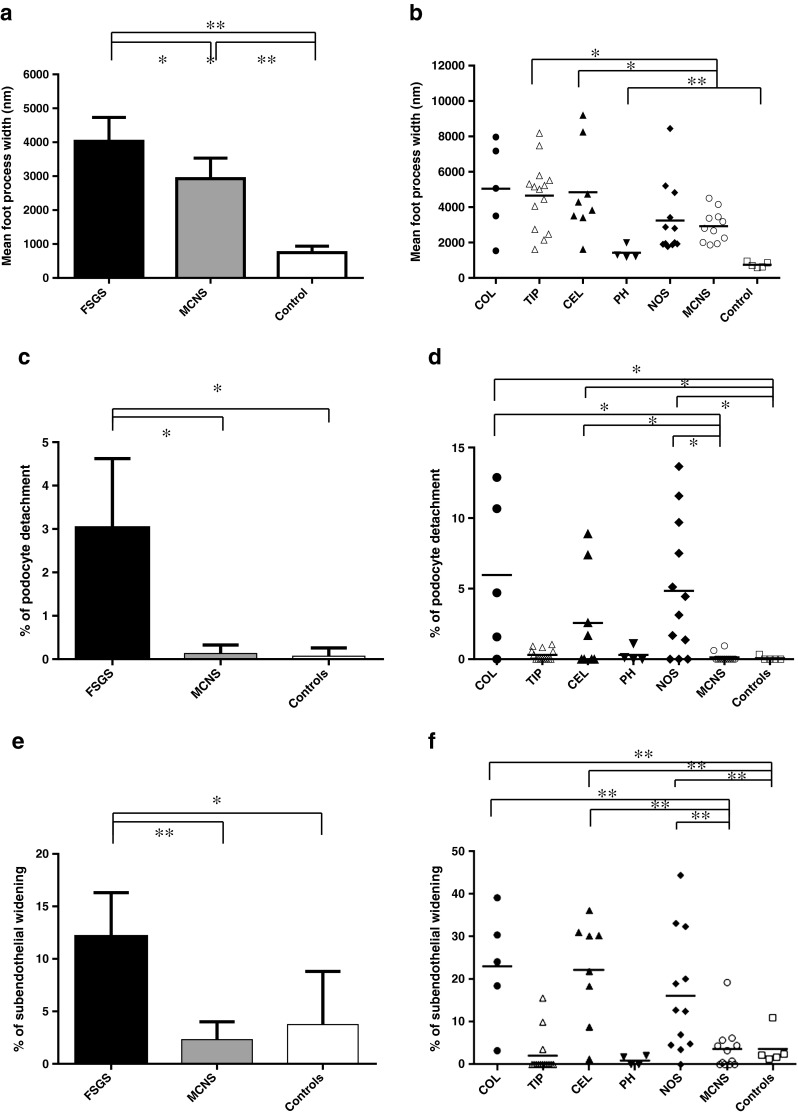
**a** Mean foot process width (**a**, **b**), percent podocyte detachment (**c**), and percent subendothelial widening (**e**), with the 95 % confidence interval in electron micrographs of the kidneys from patients with focal segmental glomerulosclerosis (*FSGS*), patients with minimal change nephrotic syndrome (*MCNS*), and control donors. **b** Mean foot process width, percent of GBM length with PD (% of podocyte detachment; **d**) and percent of GBM length with SW (% of subendothelial widening; **f**) in electron micrographs of the kidneys from patients with each variant of FSGS, patients with MCNS and control donors. *P < 0.05; **P < 0.01. *COL* collapsing variant, *TIP* tip variant, *CEL* cellular variant, *PH* perihilar variant, *NOS* not otherwise specified variant

The mean FPW was significantly higher in all groups of FSGS (4004 ± 339 nm) and, to a lesser degree, of MCNS (2926 ± 270 nm) than in that in control kidneys (747 ± 68 nm, *P* < 0.05, Fig. 3a). The mean FPW was significantly higher in patients with the COL, TIP, and CEL variants than that in patients with other variants of FSGS (*P* < 0.05, Fig. 3d), and patients with the CEL or TIP variant had a significantly higher mean FPW than patients with MCNS (*P* < 0.05, Fig. 3b). The mean FPW was lowest in the PH variant, but still significantly higher than that in controls (*P* < 0.01).

The mean % of GBM length with PD was 3.0 ± 0.8 % in patients with FSGS and 0.1 ± 0.1 % in MCNS (*P* < 0.05, Fig. 3c). No significant difference was detected between MCNS and control groups (0.1 ± 0.1 %). The mean % of GBM length with PD was higher in patients with the COL, CEL, and NOS variants than in those with other variants of FSGS, MCNS, or controls (*P* < 0.05, Fig. 3d). The mean % of GBM length with PD in patients with the TIP and PH variants was not significantly different from that in controls.

The mean % of GBM length with SW was 13.0 ± 2.4 % in patients with FSGS and 2.3 ± 1.6 % in MCNS (*P* < 0.01, Fig. 3e). No significant difference was detected between MCNS and control groups (3.7 ± 1.8 %). The mean % of GBM length with SW was higher in patients with the COL, CEL, and NOS variants than in those with other variants of FSGS (*P* < 0.05), MCNS, and controls (*P* < 0.01, Fig. 3f). Morphometric data of mean foot process width; mean percentage of podocyte detachment; or subendothelial widening in FSGS, MCNS, and control patients are also shown in Supplemental data 2.

### Relations between clinical and ultrastructural parameters in FSGS

**Fig. 4:**
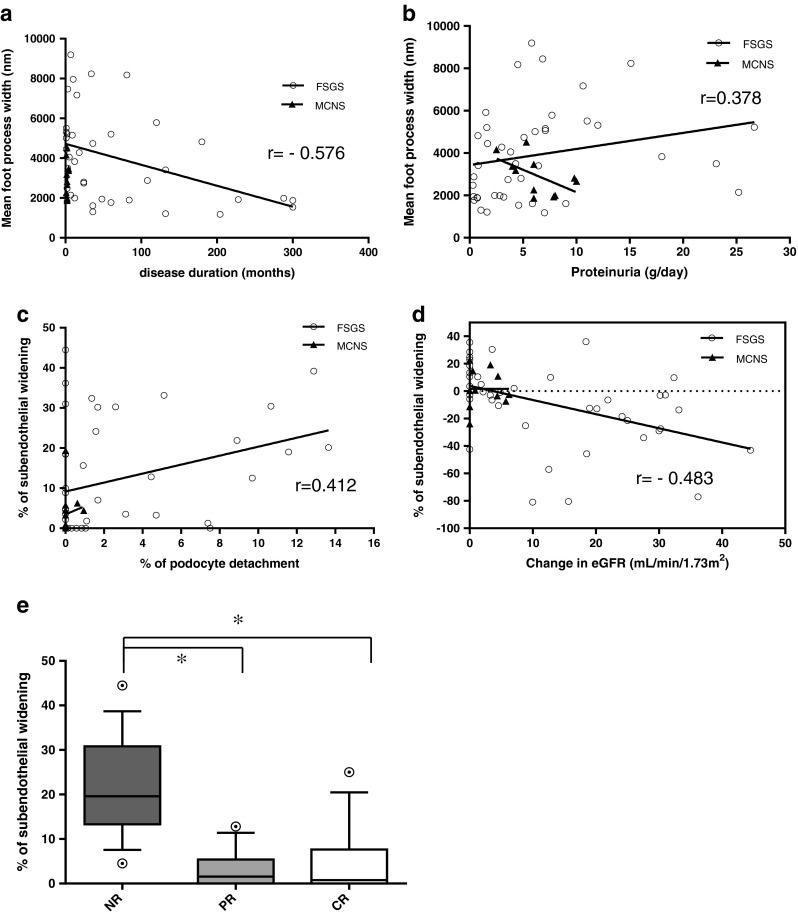
**a** Correlation between disease duration and mean foot process width in individual patients with FSGS (*white circles*) and MCNS (*black triangles*). **b** Correlation between proteinuria at the time of biopsy and mean foot process width in individual patients with FSGS (*white circles*) and MCNS (*black triangles*). **c** Correlation between percent of GBM length with podocyte detachment (% of podocyte detachment) and percent of GBM length with SW (% of subendothelial widening) in individual patients with FSGS (*white circles*) and MCNS (*black triangles*). **d** Correlation between change in eGFR and percent of GBM length with SW (% of subendothelial widening) in individual patients with FSGS (*white circles*) and MCNS (*black triangles*). **e** Percent of GBM length with SW (% of subendothelial widening) according to treatment response (*NR* non-response, *PR* partial remission, *CR* complete remission). The % of subendothelial widening was significantly increased in NR samples compared with PR or CR samples (*P < 0.05). The *horizontal line in the middle of each box* indicates the median; the *top and bottom borders of the box* mark the 75th and 25th percentiles, respectively; the *whiskers* mark the 90th and 10th percentiles; and the *circles* indicate outliers. Analysis of variance followed by the Tukey post hoc test was used for the statistical analysis

The mean FPW in the FSGS group was associated with shorter disease duration (*r* = −0.576; *P* < 0.01, Fig. 4a) and the degree of proteinuria at the time of biopsy (*r* = 0.378, *P* < 0.05, Fig. 4b). Multivariate analysis revealed that the mean FPW in the FSGS group was associated with shorter disease duration (*P* < 0.05), but not with proteinuria. No association was detected between mean FPW and other ultrastructural parameters.

The mean % of GBM length with PD in patients with FSGS correlated with longer disease duration (*r* = 0.436, *P* < 0.05) and the mean % of GBM length with SW (*r* = 0.412, *P* < 0.05, Fig. 4c). On multivariate analysis, the mean % of GBM length with PD was associated with the mean % of GBM length with SW (*P* < 0.05), but not with disease duration.

The mean % of GBM length with SW in patients with FSGS was associated with the following clinical parameters: lower eGFR (*r* = −0.413, *P* < 0.01) and proteinuria (*r* = 0.312, *P* < 0.05) at the final observation, a greater reduction in eGFR from the time of biopsy (*r* = −0.483, *P* < 0.01, Fig. 4d), and a lower remission rate (*r* = −0.530, *P* < 0.01). On multivariate analysis, the mean % of GBM length with SW was only correlated with the change in eGFR (*P* < 0.05). Figure 4e shows the distribution of % of GBM length with SW according to treatment response. The median % of GBM with SW was 19.6 in patients with NR, 15 in PR, and 0.7 in CR (NR vs. PR or CR; *P* < 0.05; Fig. 4e), and the median % of GBM length with SW in NR was significantly higher than in PR or CR. There was no statistical difference between CR and PR.

## Discussion

The highly specialized podocyte plays a major role in the glomerular filtration barrier. The podocyte foot process and its associated slit membrane are finely organized structures that maintain the filtration barrier, and their structural loss, represented by foot process effacement, is considered a good indicator of proteinuric glomerular disease. A few morphometric studies have been performed to quantify foot process effacement with the FPW parameter, and they have suggested that the degree of FPW may be related to the underlying renal disease [5, 31]. FPW in patients with MCNS was higher than that in patients with proteinuric IgA nephropathy [31] and was more extensive in patients with FSGS than those with MCNS [5]. Our finding, that mean FPW in patients with FSGS is significantly higher than that in patients with MCNS, supports previously reported data. These findings indicate that quantitative analysis of FPW might be useful to distinguish between FSGS and MCNS, particularly when a biopsy specimen does not contain an adequate number of glomeruli.

The mean FPW in patients with FSGS was significantly higher in the COL, CEL, and TIP variants than that in the PH variant, with the NOS variant in between. The segmental lesions in patients with the COL, CEL, and TIP variants are histologically active with hypercellularity, caused by proliferation of intrinsic glomerular cells or infiltrating inflammatory cells [3, 24], whereas segmental lesions in the PH and NOS variants are characterized histologically by sclerotic lesions that show an increase in extracellular matrix and hyaline deposits. Such pathological aspects are supported by clinical data, in that patients with the COL, CEL, and TIP variants developed severe proteinuria and had shorter disease duration. Thus, the higher mean FPW in patients with the COL, CEL, and TIP variants might represent more severe acute-phase podocyte injury, whereas the lower mean FPW in patients with the PH variant suggests less severe podocyte injury.

Differentiated podocytes are endowed with a limited potential to divide [8]. As has been suggested by Kriz et al., in an ablation model of FSGS, tuft hypertrophy in response to the reduced number of functioning nephrons does not increase podocyte cell number, and podocytes are forced to change their appearance, such as foot process effacement, cell body attenuation, and finally detachment from the GBM. Repair of such defects is only possible by hypertrophy of adjacent intact podocytes, which is quite limited [14]. In our study, patients in the FSGS group demonstrated a significantly higher % of GBM length with PD than the control group, but not patients in the MCNS group. PD has been reported in patients with several renal diseases, including FSGS, diabetic nephropathy, amyloidosis, and reflex nephropathy [9, 12, 28, 30]; however, such findings have rarely been observed in patients with MCNS [15]. Our findings are consistent with the previously reported results. The exact mechanisms responsible for PD in patients with FSGS but not in those with MCNS remain unclear. Recent studies have shown that dendrin, a protein that promotes apoptosis of podocytes, is located in the cytoplasm of normal podocytes and in the kidneys of patients with MCNS [6], whereas it translocates to the nucleus in patients with FSGS [1]. These results suggest that regulation of podocyte homeostasis and apoptosis in FSGS is different from that in MCNS. Furthermore, we found the % of GBM length with PD associated with longer disease duration, suggesting that PD is an irreversible event, reflecting severity and disease duration of FSGS. Taken together, podocyte injury culminates in detachment from GBM, which may represent an end-stage manifestation of podocytopathy in patients with FSGS.

SW is a morphological feature indicative of endothelial injury. We found in the FSGS group a significantly higher % of GBM length with SW than in the control group. Endothelial dysfunction has been recently observed in patients with FSGS. In the Columbia classification, the CEL variant is defined by the presence of endocapillary hypercellularity, which consists of infiltrating foam cells and/or other inflammatory cells together with swollen endothelial cells. These histological features reflect endothelial injury. A recent report has suggested that endothelial dysfunction in FSGS, which involves increased levels of endothelial cell-derived circulating VCAM-1 and thrombomodulin levels, is largely related to disease activity [33]. Moreover, in an experimental model of glomerulosclerosis, a reduced number of endothelial cells were found, due to endothelial apoptosis [13]. These findings suggest that endothelial injury is involved in the development of FSGS. We excluded cases with secondary FSGS from analysis. However, we observed in cases with secondary FSGS induced by calcineurin inhibitors after renal transplantation that endothelial injury is more prominent than podocyte injury (unpublished data). Further studies are needed to elucidate the involvement of endothelial injury in the development of secondary FSGS.

Coexistence of podocyte and endothelial injury has been reported in several human [22] and experimental renal diseases [4, 13, 27]. A rat experimental model of endothelial injury has demonstrated thickening of capillary walls from SW and podocyte injury [23], which later resulted in glomerulosclerosis [2]. In our study, the mean % of GBM length with PD correlated with the mean % of GBM length with SW in the FSGS group, and this was consistent with in vivo experimental studies in a remnant kidney model [13, 27]. Furthermore, both parameters were higher in patients with the COL, CEL, or NOS variants, and the mean % of GBM length with SW was associated with a subsequent reduction in eGFR and a lower remission rate at the time of final observation. The median % of subendothelial widening was also significantly increased in NR samples compared with PR or CR samples. The 75th percentile of % of GBM length with SW was slightly higher in CR than in PR in our study. However, its median and 25th percentile was lower in CR than in PR, and between PR and CR the differences were not significant. These findings indicate that PD and SW are important factors in progression of FSGS, and both parameters might be prognostic determinants.

Recent studies have suggested that crosstalk between podocytes and endothelial cells may lead to glomerulosclerosis [4, 7]. Eremina et al. have established a murine model in which vascular endothelial growth factor (VEGF) production in podocytes is deleted [7]. These mice show obliterated/collapsed glomerular capillaries with swollen endothelial cells, which suggests that VEGF production by podocytes is required for homeostasis of adjacent glomerular endothelium. Moreover, Daehn et al. induced experimental glomerulosclerosis in which endothelin-1 and its receptor are responsible for mitochondrial dysfunction, with PD as a result of crosstalk between podocytes and endothelial cells, which was mitigated by preventing mitochondrial oxidative stress in endothelial cells [4]. Endothelial damage can occur because of podocyte injury and plays a role in the development of glomerulosclerosis in FSGS.

Based on its etiology, primary FSGS can be classified as immunologic or genetic. Crosstalk between podocytes and endothelial cells in genetic FSGS has not been well studied. Electron microscopy of renal biopsies of multiple members of two families with collapsing FSGS showed extensive wrinkling of the GBM, with extensive foot process effacement and hypertrophy [18]. Irregularly aggregated electron-dense material in podocyte cytoplasm has been reported as distinctive ultrastructural feature of podocyte injury in *ACTN4* biopsies [10]. Further research is required to provide evidence of endothelial injury in genetic FSGS.

In conclusion, we evaluated electron microscopic parameters of FPW and PD as markers of podocyte injury and SW as a marker of endothelial injury. Quantitative measurement of FPW may be useful to distinguish between FSGS and MCNS and to evaluate histological activity in FSGS. Compared with FPW, PD is a phenomenon more specific for FSGS, representing a more severe and later manifestation of podocyte injury. SW occurs also in patients with FSGS, and quantitative evaluation of SW and PD might provide indicators of prognosis in patients with FSGS. Further studies are needed to evaluate their possible impact on the disease progression of FSGS.

## Electronic supplementary material

Supplementary data 1 (DOCX 67.5 kb)

Supplementary data 2 (DOCX 14.6 kb)
