# Supplementary material for: Podocyte and endothelial injury in focal segmental glomerulosclerosis: an ultrastructural analysis
Source: Virchows Arch. 2015 Aug 13;467(4):449–58. doi: 10.1007/s00428-015-1821-9 (PMC4609310; doi:10.1007/s00428-015-1821-9)
Supplement: Supplementary file 1 — Supplementary data 1 (DOCX 67.5 kb) [file 428_2015_1821_MOESM1_ESM.docx]

Supplemental data 1

Morphometric data of mean foot process width, mean percentage of podocyte detachment or subendothelial widening in FSGS, MCNS and control

*

*
