# Supplementary material for: Podocyte and endothelial injury in focal segmental glomerulosclerosis: an ultrastructural analysis
Source: Virchows Arch. 2015 Aug 13;467(4):449–58. doi: 10.1007/s00428-015-1821-9 (PMC4609310; doi:10.1007/s00428-015-1821-9)
Supplement: Supplementary file 2 — Supplementary data 2 (DOCX 14.6 kb) [file 428_2015_1821_MOESM2_ESM.docx]

Supplemental data 2 eGFR changes in patients with FSGS and MCNS

FSGS MCNS Controls p^a^

All COL TIP CEL PH NOS

Case number 43 5 (11.6 %) 14 (32.6%) 8 (18.6%) 4 (9.3%) 12 (27.9%) 11 5

Foot process width (nm) 4004 ± 339 5047 ± 1177 4541 ± 476 4850 ± 908 1424 ± 192 3239 ± 574 2926 ± 270 747 ± 68 <0.05

Range (nm ) 1181−9196  1534−7965 1616–8180 1611−9196 1181–1996 1773−8346 1863−4509 589−949

Podocyte detachment (%) 3.0 ± 0.8 6.7 ± 3.0 0.3 ± 0.1 2.6 ± 1.3 0.3 ± 0.3 5.9 ± 2.0 0.1 ± 0.1 0.1 ± 0.1 <0.05

Subendothelial widening (%) 13.0 ± 2.4 24.6 ± 11.0 2.1 ± 1.3 22.3 ± 4.3 1.0 ± 0.6 17.0 ± 4.4 2.3 ± 1.6 3.7 ± 1.8 <0.01

COL, collapsing variant; TIP, tip variant; CEL, cellular variant; PH, perihilar variant; NOS, not otherwise specified variant; MCNS, minimal change nephrotic syndrome; NS, not significant. Quantitative variables are mean ± standard error; 　^a^ Analysis of variance between all FSGS vs MCNS, Student t test.

Legend

Supplemental data 1：The changes in the estimated glomerular filtration rate (eGFR) between initial and final observation in patients with each variant of FSGS and MCNS. *P<0.05. COL, collapsing variant; TIP, Tip variant; CEL, cellular variant; PH, perihilar variant; NOS, not otherwise specified variant.

Supplemental data 2：Morphometric data of mean foot process width, mean percentage of podocyte detachment or subendothelial widening in FSGS, MCNS and control patients.
